# A Pilot Proof-Of-Principle Analysis Demonstrating Dielectrophoresis (DEP) as a Glioblastoma Biomarker Platform

**DOI:** 10.1038/s41598-019-46311-8

**Published:** 2019-07-16

**Authors:** Jean Lewis, Ali A. Alattar, Johnny Akers, Bob S. Carter, Michael Heller, Clark C. Chen

**Affiliations:** 1 Department of Nanoengineering, University of California San Diego, La Jolla, CA USA; 2 School of Medicine, University of California San Diego, La Jolla, CA USA; 3 Department of Neurosurgery, University of California San Diego, La Jolla, CA USA; 4 Department of Neurosurgery, Massachusetts General Hospital, Boston, MA USA; 5 Department of Neurosurgery, University of Minnesota, Minneapolis, MN USA

**Keywords:** Diagnostic markers, CNS cancer, CNS cancer

## Abstract

Extracellular vesicles (EVs) are small, membrane-bound particles released by all cells that have emerged as an attractive biomarker platform. We study the utility of a dielectrophoretic (DEP) micro-chip device for isolation and characterization of EVs derived from plasma specimens from patients with brain tumors. EVs were isolated by DEP chip and subjected to on-chip immunofluorescence (IF) staining to determine the concentration of glial fibrillary acidic protein (GFAP) and Tau. EVs were analyzed from the plasma samples isolated from independent patient cohorts. Glioblastoma cell lines secrete EVs enriched for GFAP and Tau. These EVs can be efficiently isolated using the DEP platform. Application of DEP to clinical plasma samples afforded discrimination of plasma derived from brain tumor patients relative to those derived from patients without history of brain cancer. Sixty-five percent (11/17) of brain tumor patients showed higher EV-GFAP than the maximum observed in controls. Ninety-four percent (16/17) of tumor patients showed higher EV-Tau than the maximum observed in controls. These discrimination thresholds were applied to plasma isolated from a second, independent cohort of 15 glioblastoma patients and 8 controls. For EV-GFAP, we observed 93% sensitivity, 38% specificity, 74% PPV, 75% NPV, and AUC of 0.65; for EV-Tau, we found 67% sensitivity, 75% specificity 83% PPV, 55% NPV, and AUC of 0.71 for glioblastoma diagnosis. This proof-of-principle study provides support for DEP-IF of plasma EVs for diagnosis of glioblastoma.

## Introduction

In many forms of cancer, early detection and diagnosis have led to improved survival^1^. Early detection affords opportunities for more complete surgical resection of neoplastic tissue^2^, as well as treatment of cancer cells before they acquire a complex mutational landscape and intra-tumoral heterogeneity^3–5^, both of which remain major challenges to meaningful therapeutic response^6^. Unfortunately, such early detection is currently impossible for glioblastoma, the most common form of adult primary brain cancer^7^. By the time of clinical presentation, tumors are typically large and the glioblastoma cells often exhibit complex intra-tumoral heterogeneity^3,4,8^. As such, early glioblastoma detection remains an unmet need in neuro-oncology.

**Figure 1:**
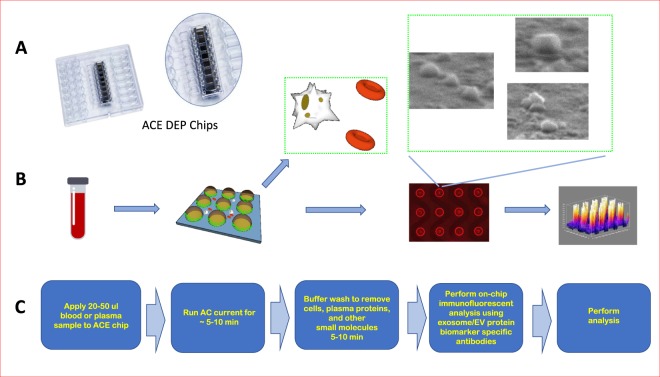
Overview of DEP chip, isolated extracellular vesicles, and work-flow. (**A**) image of the chip with magnified image in circle. (**B**) Schematic representing work-flow for isolation of EVs, beginning with an undiluted sample of patient plasma which is applied to the DEP chip. Cells and large debris collect in between electrodes and are removed. Extracellular vesicles and similarly sized nanoparticles accumulate at electrode edges and can be detected via electron microscopy or immunofluorescence staining of their contents. SEM pictures of extracellular vesicles were taken on 3-30-2018 by Juan Pablo Hinestrosa, at Biological Dynamics, Inc., San Diego, CA and used with permission. (**C**) Flow-diagram with text description of work-flow.

There is emerging evidence supporting extracellular vesicles (EVs) as a promising biomarker platform for glioblastomas. EVs are small, membrane-bound particles composed of lipids and proteins that range from 50 to 4000 nm in size^9^. These vesicles normally support cell-to-cell communication, mediate the export of cellular contents, and modulate membrane morphology^10^. Importantly, glioblastoma cells secrete EVs containing tumor-specific microRNA^9,11,12^, mRNA^13^, and proteins that can be detected in peripheral blood^14^. To further develop EVs as a biomarker platform, we have developed a highly efficient, single-step dielectrophoretic (DEP) separation method for EV isolation and analysis^15^. In brief, DEP uses an alternating current to generate a separation force that attracts particles of different sizes to distinct regions between chip electrodes^16^. We have optimized DEP microarray chips to isolate EVs from patient plasma samples and developed a method for on-chip immunofluorescence analysis for quantification of protein content (Fig. 1)^17^.

Here we tested this platform to detect EV-contained glial fibrillary acidic protein (GFAP) and Tau in patients with and without a diagnosis of glioblastoma. GFAP and Tau are proteins that are highly expressed in many cell types in the central nervous system (CNS)^18,19^. GFAP encodes an intermediate filament protein that is highly abundant in astrocytes^18^, and Tau is a microtubule-stabilizing protein that is highly expressed in neurons^19^. Both proteins closely associate with cellular membranes and can be detected in EVs^20–22^. Importantly, these proteins are present at extremely low levels in the peripheral blood of patients without CNS injury but are released after CNS injury, causing a detectable elevation in serum concentration^20,23,24^. We hypothesized that GFAP and Tau had potential as biomarkers for glioblastoma because 1) glioblastoma growth inevitably damages nearby astrocytes and neurons, releasing free GFAP and Tau into the surrounding tissues and fluid compartments^25^, and 2) GFAP and Tau are highly expressed in glioblastoma and may be enriched in their EVs^26,27^. As such, EVs isolated from plasma of brain tumor patients should contain higher concentrations of GFAP and Tau compared to samples from normal patients. Our findings support this hypothesis and provide proof-of-principle data for DEP microarray and on-chip immunofluorescence analysis of EV-contained GFAP and Tau as a potential glioblastoma detection platform.

## Results

### Measurement of GFAP and Tau in cultured tumor cells and EVs

**Figure 2:**
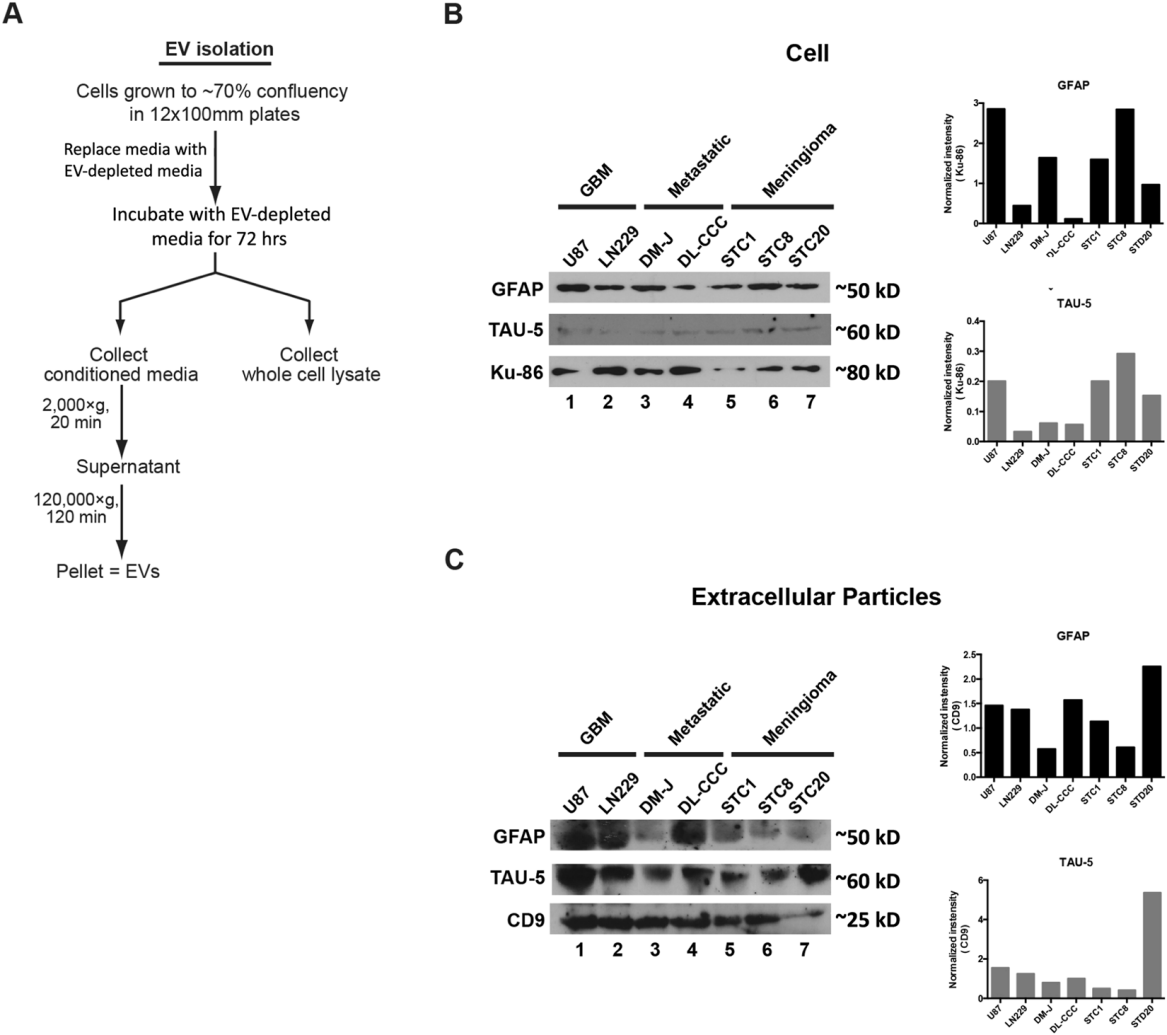
Protocol schematic and Western blot results of analysis of cultured cell lines. Western blots were repeated at least twice. Representative images are shown. (**A**) Schematic representation of protocol used for the isolation of extracellular vesicles from conditioned media. (**B**) GFAP and TAU-5 level in tumor, metastatic and benign cell lines were analyzed with Western blotting. Each row is derived from the same gel, which was cut based on molecular weight range. The right panel shows densitometric analysis of GFAP and TAU-5 normalized to the loading control Ku-86. (**C**) GFAP and TAU-5 level in EVs secreted by tumor, metastatic and benign cell lines were analyzed with Western blotting. Each row is derived from the same gel, which was cut based on molecular weight range. The right panel shows densitometric analysis of GFAP and TAU-5 normalized to the loading control CD9.

As a first step toward determining whether GFAP and Tau are candidate EV biomarkers for tumor detection, we tested whether these proteins were detectable in EVs isolated from various brain tumor cell lines. To this end, we isolated EVs from two glioblastoma cell lines (U87 and LN229), two patient-derived brain metastasis cell lines (DM-J and DL-CCC), and three patient-derived meningioma cell lines (STC1, STC8, and STC20). We assessed the relative abundance of GFAP and Tau in both whole-cell lysate and EV lysates (isolated as described in Fig. 2A) using immunoblotting. In whole-cell lysates, we detected GFAP and Tau in all cell lines tested (Fig. 2B). Similarly, we were able to detect both GFAP and Tau in all EV lysates (Fig. 2C). Interestingly, EV lysate appeared to be enriched for the Tau protein relative to the whole cell lysate. These results suggest that brain tumor cells release EVs containing GFAP and Tau and suggest that EV GFAP and Tau have potential as brain tumor biomarkers.

### DEP isolation of EVs from clinical plasma: controlled experiments

**Figure 3:**
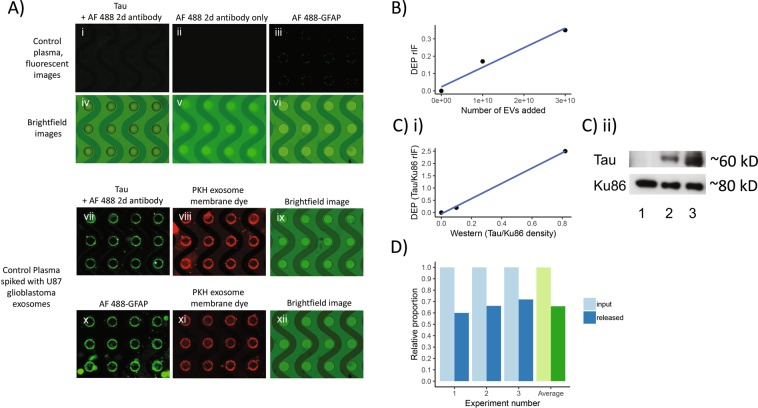
(**A**) Images of DEP microchips run with control plasma, with and without U87 glioblastoma exosome spike, analyzed by immunofluorescence for GFAP and Tau. (i–vi) As a control, plasma from subjects without history of brain cancer was run on DEP chips, permeabilized with saponin, and incubated either with antibodies against Tau, followed by Alexa Fluor 488 conjugated secondary antibody (i and iv), Alexa Fluor 488 conjugated secondary antibody alone (ii and v), or with directly conjugated Alexa Fluor 488 GFAP antibodies (iii and vi). The above analyzed plasma was spiked with U87 glioblastoma EVs that had been labeled with the red-fluorescent dye PKH. Samples were run on two separate DEP chips, saponin-permeabilized, and incubated either with antibodies against Tau, followed by Alexa Fluor 488 conjugated secondary antibody (vii and ix), or with directly conjugated Alexa Fluor 488 GFAP antibody (x and xii). Panels vii and x show illumination of Alexa Fluor 488-labeled antibodies in the green channel; panels viii and xi show the corresponding images illuminated in the red channel for PKH dye; panels ix and xii are the corresponding brightfield images. (**B**) GFAP immunofluorescence of EV-depleted media as glioblastoma U87-derived EVs are added back to the media. There is a linear, dose-dependent increase in GFAP IF as EVs are added. (**C**) Correlation between Western blot-derived Tau IF and DEP-derived Tau IF for solutions of 0, 10, and 50 mcg of EVs. (i) Both axes represent Tau IF normalized to the Ku86. There is a direct correlation between Western blot-measured and DEP-measured Tau IF. (ii) A representative Western blot from which the densitometric analysis in (**C**) (i) is derived. Columns 1, 2, and 3, represent 0, 10, and 50 mcg of EVs, respectively. (**D**) Efficiency of EV capture using DEP. Known quantities of EVs were added to solution then isolated on the DEP chip. EVs were then released and quantitated. The proportion of input EVs that were eventually released and quantitated was regarded as the capture efficiency. The experiment was repeated three times (paired blue columns) and the average of three iterations is shown as the pair of green columns.

To demonstrate that glioblastoma derived EVs can be isolated using the DEP platform, we performed the following experiments. We first isolated EVs from plasma from a subject without history of brain cancer using DEP-IF. Immunofluorescence staining for Tau and GFAP on these isolated EVs indicated minimal to no signal (Fig. 3Ai,iii). Plasma from the same subject was then spiked with EVs derived from the U87 glioblastoma cell line and labelled with the fluorescent dye PKH67^28^. DEP-isolated EVs from this spiked plasma showed avid Tau (Fig. 3Avii) and GFAP (Fig. 3Ax) staining. As expected, the DEP-isolated EVs also showed high PKH signal (Fig. 3Aviii,xi). Moreover, we saw a dose-dependent increase in GFAP-IF as more EVs were added back to the plasma (Fig. 3B). These results, together with our previous publication demonstrating that DEP-isolated particles stained positive for CD63 and TSG 101^15^, suggest that U87-derived PKH-labelled EVs were retained on the DEP chip and support the utility of DEP as an EV isolation platform.

Next, we determined the correlation between the DEP-detected Tau IF with the input EV Tau levels, quantitated using immunoblotting. Three different quantities of U87-derived EVs were spiked into the plasma. Prior to spiking, half of the sample was lysed and immunoblotted for Tau protein (Fig. 3Cii). The other half was loaded onto the DEP chip. The level of Tau IF was determined after DEP-EV isolation. We found a good correlation between Western blot-detected Tau and DEP-detected Tau IF (Fig. 3Ci).

Finally, we determined the efficiency of EV capture by DEP. In this experiment, we loaded ~1.0 × 10^10^ U87 EVs into the control plasma. After DEP, DEP-isolated EVs were released from the chip and quantitated. Percent capture was calculated by comparing the input EV number to the released EV number. We estimated that the DEP platform captured at least 60–70% of the input EV. This result is now indicated as Fig. 3D.

### DEP isolation of EVs from clinical plasma: clinical correlation

We next analyzed plasma derived from patients diagnosed with meningioma, brain metastasis, and glioblastoma and compared them to normal controls. For isolation of EVs, 30–50 microliters of undiluted patient plasma were loaded onto the DEP chip, an alternating current electric field was applied for 10 minutes to isolate EVs, serial washes were performed, and cells were permeabilized for subsequent on-chip immunofluorescence assessment as described previously^15^. The time to complete the process was 30 minutes (Fig. 1).

**Figure 4:**
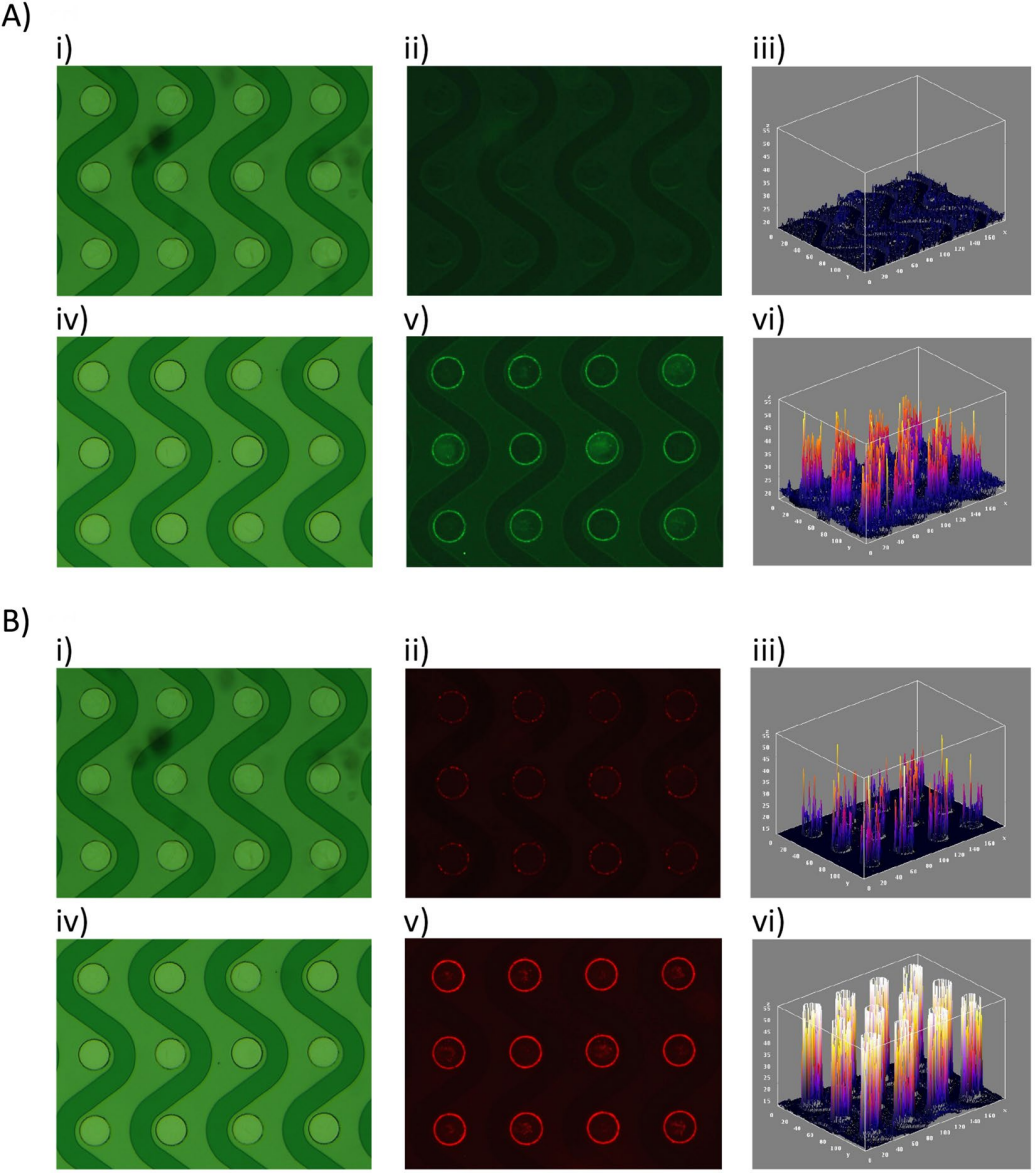
Images of DEP micro-chip loaded with control plasma and with brain tumor patient plasma and analyzed with immunofluorescence for GFAP and Tau. (**A**) DEP-isolated extracellular vesicles from brain cancer patient plasma (bottom row), but not normal plasma (top row), contain GFAP. Plasma from a healthy donor (panels i–iii) or from a brain cancer patient (panels iv–vi) were each applied to a DEP chip. EVs immobilized onto the DEP chip were permeabilized with saponin, then positively labeled with Alexa Fluor 488-conjugated anti-GFAP antibodies and visualized directly. Panels i and iv show the brightfield images for each chip; panels ii and v are the corresponding fluorescent images, and panels iii and vi are direct 3D projections for panels ii and v, showing relative quantitation. (**B**) DEP-isolated EVs from brain cancer patient plasma (bottom row), but not normal plasma (top row), contain high levels of Tau. Plasma from a healthy donor (panels i–iii) or from a brain cancer patient (panels iv–vi) were each applied to a DEP chip. EVs immobilized onto the DEP chip were permeabilized with saponin, then positively labeled with anti-tau antibodies and visualized with Alexa Fluor 594-conjugated secondary antibody (red). Panels i and iv show the brightfield images for each chip; panels ii and v are the corresponding fluorescent images, and panels iii and vi are direct 3D projections for panels ii and v, showing relative quantitation.

**Figure 5:**
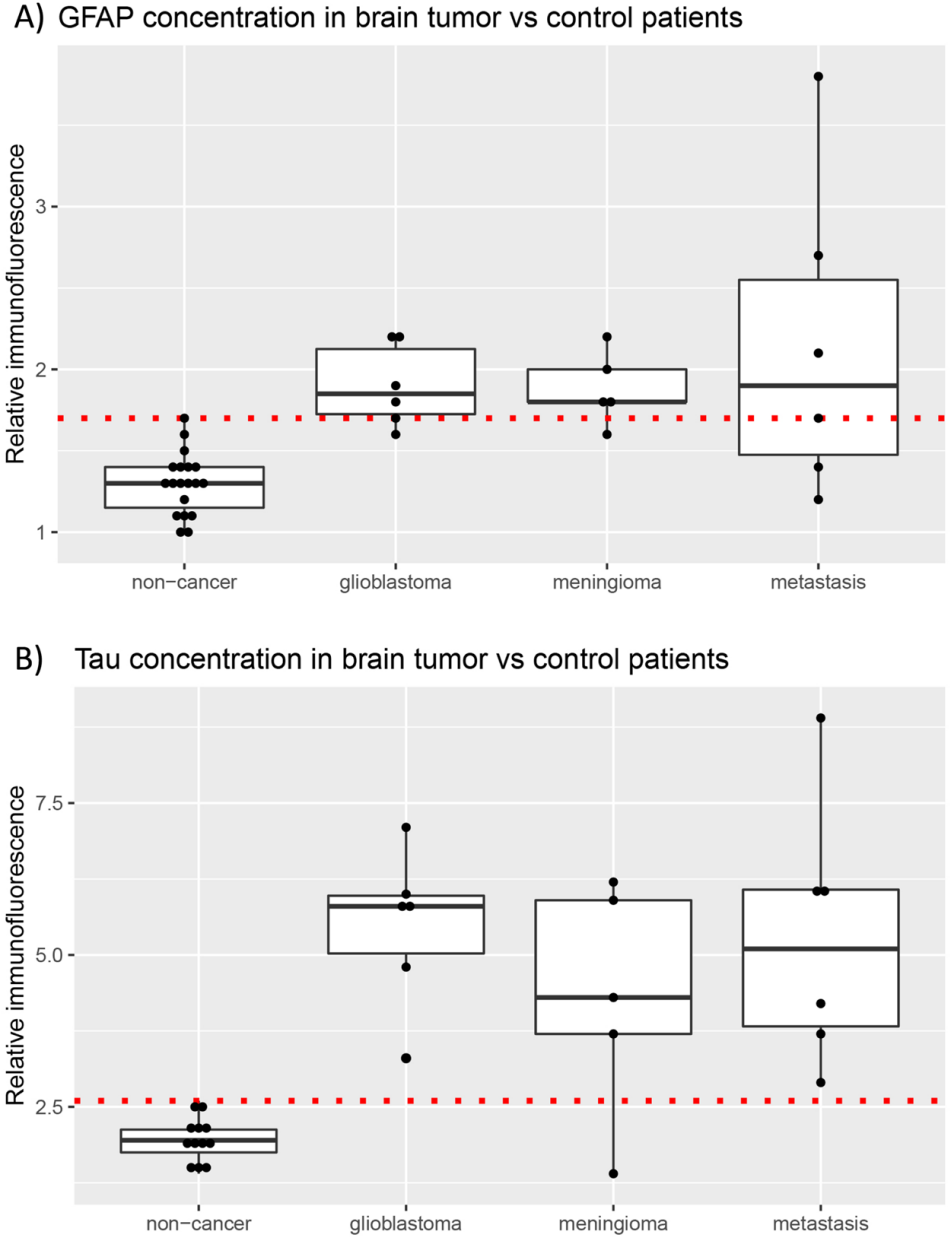
Box plots demonstrating distribution of biomarker immunofluorescence in the initial experimental cohort, stratified by tumor type. (**A**) GFAP: The red dotted horizontal line demarcates the discrimination threshold of 1.7 relative immunofluorescence units. Eleven of 17 tumor samples (65%) exceeded this threshold. (**B**) Tau: The red dotted horizontal line demarcates the discrimination threshold of 2.6 relative immunofluorescence units. Sixteen of 17 tumor samples (94%) exceeded this threshold.

We collected plasma from 17 patients with a tissue-confirmed diagnosis of glioblastoma (n = 6), metastasis (n = 5), or meningioma (n = 6) and 23 non-tumor controls. Representative brightfield, qualitative fluorescent, and quantitative fluorescent images are shown in Fig. 4. Qualitative analysis of the chip demonstrated increased fluorescence over all electrodes in samples from brain-tumor patients (Fig. 4A,B panel v) compared to non-tumor controls (Fig. 4A,B panel ii). Quantitative assessment of GFAP and Tau IF are shown as box plots in Fig. 5. None of the normal patient plasma samples demonstrated >1.7 rIF units for GFAP (Fig. 5A) or >2.6 relative immunofluorescence units (rIF) for Tau (Fig. 5B). In contrast, 16/17 (94%) and 11/17 (65%) brain tumor patients had Tau and GFAP rIF above these values, respectively. We did not detect significant differences in GFAP or Tau concentration between patients with meningioma, brain metastasis, or glioblastoma (Fig. 5A,B).

### Validation in an independent patient cohort

**Figure 6:**
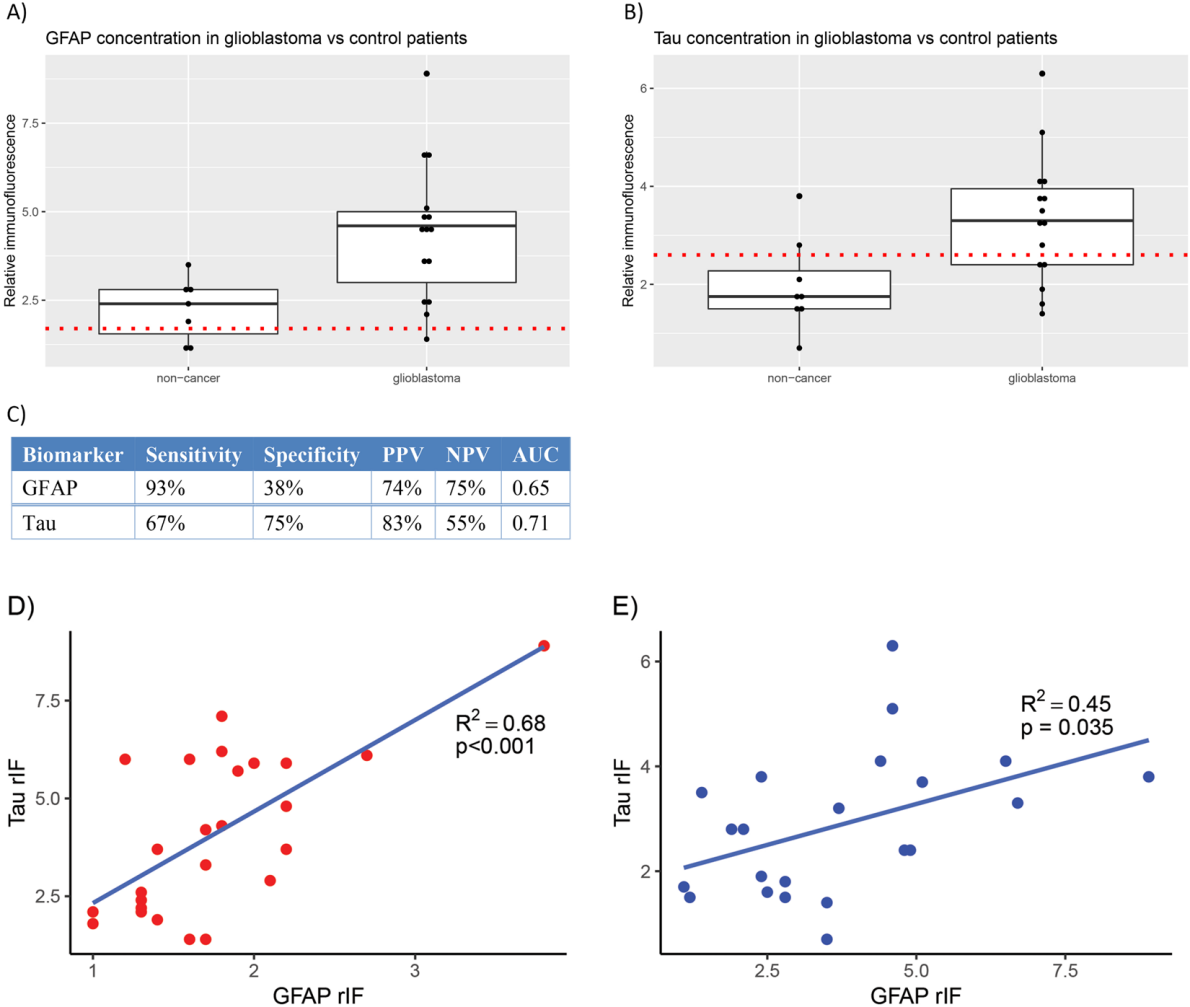
Box plots demonstrating distribution of biomarker immunofluorescence in the validation cohort, stratified by diagnosis of glioblastoma. (**A**) GFAP: The red dotted horizontal line demarcates the previously determined discrimination threshold of 1.7 relative immunofluorescence units. (**B**) Tau: The red dotted horizontal line demarcates the previously determined discrimination threshold of 2.6 relative immunofluorescence units. (**C**) Model performance measures for diagnosis of glioblastoma. (**D**) Scatter plot demonstrating correlation between GFAP and Tau rIF in the initial cohort. (**E**) Scatter plot demonstrating correlation between GFAP and Tau rIF in the validation cohort.

To determine the diagnostic value of the discrimination threshold established in our exploratory cohort (Tau > 2.6 rIF and GFAP > 1.7 rIF), we analyzed prospectively collected plasma from a cohort of 15 glioblastoma patients and 8 control subjects without history of brain tumor (Fig. 6A,B). EV GFAP fluorescence exceeding 1.7 rIF had a sensitivity of 93% and specificity of 38% for diagnosis of glioblastoma. The positive and negative predictive values were 74% and 75%, respectively. The area under the receiver operating characteristic curve (AUC) was 0.65. For Tau, the sensitivity, specificity, PPV, NPV, and AUC were: 67%, 75%, 83%, 55%, and 0.71 (Table 1, Fig. 6C). These values are on par with those published in pilot biomarker studies, supporting the utility of DEP-IF as a plausible biomarker platform^12,29^.

**Table 1:** Statistical performance measures for each biomarker in validation cohort.

Biomarker	Sensitivity	Specificity	PPV	NPV	AUC
GFAP	93%	38%	74%	75%	0.65
Tau	67%	75%	83%	55%	0.71

### Correlation between EV GFAP and Tau

Next, we performed Pearson correlation analysis to determine whether DEP-IF detected EV GFAP correlated with EV Tau. The correlation coefficient between EV GFAP and Tau signal was 0.68 (p < 0.001) in the initial cohort (Fig. 6D). In the validation cohort, the correlation coefficient between EV GFAP and Tau signal was 0.45 (p = 0.035) (Fig. 6E). These results suggest that EV-GFAP and Tau serve as biomarkers of redundant or related pathophysiologic processes.

## Discussion

Our study indicates that cell lines derived from glioblastoma, meningioma, and brain metastasis secrete EVs enriched for GFAP and Tau proteins. Using an on-chip DEP-IF platform, we showed that the concentration of GFAP and Tau in EVs isolated from the plasma of glioblastoma patients is higher than in patients without history of brain cancer. We derived discriminating thresholds from an initial cohort and validated these thresholds in an independent cohort of glioblastoma and non-tumor subjects. Diagnostic test performance was within the acceptable range for a pilot, proof-of-principle study^11,29^. We have provided a proof-of-concept study supporting the utility of an on-chip DEP-IF platform as a blood-based diagnostic test for glioblastoma detection.

There are several major benefits to the DEP-IF platform. First, a small volume (30–50 ul) of plasma is required for the analysis. This amount of blood can be found as left-over in blood drawn in the emergency room for laboratory tests routinely performed on all patients. Second, no special processing is required beyond centrifugation to isolate plasma. Third, the platform affords highly time-efficient analysis of the samples relative to other EV based platforms. Traditional methods of EV isolation, such as ultracentrifugation, require >5 hours of preparation. Similarly, other commercial kits require preparation on the scale of hours. In contrast, the DEP-IF platform requires 45–90 minutes, from beginning to end. This time can further be reduced through antibody optimization, automation and electric field optimization. Fourth, the DEP-IF platform is very simple and requires minimal sample handling, which reduces the likelihood of sample contamination. Finally, the platform can easily be expanded into multiplex IF to simultaneously assess biomarkers other than GFAP and Tau in a single setting.

While GFAP and Tau are highly abundant in EVs derived from glioblastoma cell lines, they are unlikely to be specific biomarkers of glioblastoma. This can be seen in our experiments, where comparable concentrations of these biomarkers were also found in EVs derived from plasma of patients with meningioma and metastases. Additionally, elevated GFAP and Tau has been reported in the plasma of patients who suffered from trauma, stroke, and Alzheimer’s disease^20,30,31^. In this context, it is highly likely that injury to the brain secondary to mass effect of a growing tumor contributes to the elevation in plasma EV-contained GFAP and Tau. Further investigations are needed to dissect the relative contribution of GFAP and Tau released from adjacent tissue injury versus direct tumor release. The lack of specificity of GFAP and Tau for glioblastoma emphasizes the need for proteomic profiling of the EVs isolated from these patients, with the goal of identifying glioblastoma-specific biomarkers^32^. That said, elevated EV-contained GFAP and Tau in peripheral blood may provide an indication for additional neurologic work-up and brain imaging.

Our results raise several important questions. First, while GFAP and Tau are elevated in patients who present with glioblastoma, all these patients are afflicted with sizable tumor by the time of presentation. It remains unclear whether the sensitivity and specificity of GFAP and Tau are sufficient to afford early detection before clinical diagnosis. Second, others have shown that elevated non-EV GFAP and Tau in peripheral blood can serve as a proxy for neurologic injury^20,23,24^. The relative merits of EV and non-EV GFAP as a biomarker require further exploration. Because this study is focused on feasibility, we selected threshold values (maximum observed values in the control patients) that we consider intuitive and simple. Data derived from this study will provide the basis for power calculation in terms of the number of samples needed to afford statistical optimization of threshold discrimination value. Finally, the intriguing observation that glioblastoma secreted EVs are enriched for GFAP and Tau suggests an interesting biology that warrants further exploration. These issues, however, do not diminish the importance of our finding supporting the utility of a DEP-IF technology platform as a detection platform for glioblastoma.

## Methods

All experimental protocols were approved by the University of California San Diego Institutional Review Board under IRB 120345X. All methods were carried out in accordance with relevant guidelines and regulations. Informed consent was obtained from all subjects or from a parent or legal guardian if subjects were younger than 18.

### EV free media preparation

EV-depleted medium was prepared by ultracentrifugation of DMEM supplemented with 20% FBS at 120,000 × g for 18 hours at 4 °C. The medium was then diluted to a final concentration of 10% FBS and used to culture cell lines as described.

### Cell lines and cell culture

Two human glioblastoma cell lines (U87MG and LN229), two metastatic cell lines (DM-J and DL-CCC), and three meningioma cell lines (STC1, STC8, and STC20) were cultured in DMEM supplemented with 10% FBS. At 60–70% confluency, the standard culture medium was replaced with EV depleted medium. The cells were cultured for an additional 72 hours before EV collection from the cell-free supernatants.

### Extracellular vesicle (EV) isolation by ultracentrifugation

EVs were prepared as previously described^11^. Conditioned media was first centrifuged at 2,000 × g for 20 minutes to remove cellular debris. The resultant supernatant was then transferred to ultracentrifuge tubes for ultracentrifugation at 120,000 × g for 2 hours. The supernatant was discarded and the EV pellets were re-suspended in 150 μL of PBS for storage at −80 °C. All centrifugation steps were performed at 4 °C.

### Plasma samples

Plasma specimens were collected at the University of California San Diego (UCSD) Medical Center under IRB 120345X. All protocols were approved by the UCSD IRB and informed consent was signed by each patient. Blood was collected using an 18-gauge needle venipuncture into clot-activating blood collection tubes with gel separator (BD Biosciences) prior to surgical resection. Plasma sample from non-tumor controls were collected as residual samples from patients presented to the emergency room who underwent blood-draw for routine blood chemistry. Patients with presentation of head trauma were excluded from this study. The samples were processed by spinning at 1,100·g within 30 min of collection and stored at −80 °C.

### AC dielectrophoretic isolation of exosomes from plasma

AC electrokinetic microarray chips were purchased from Biological Dynamics (San Diego, CA). Fluid flow across the chip was regulated using a syringe pump set to withdrawal mode. Tygon tubing (inner diameter, 0.020 inches; outer diameter, 0.060 inches) was attached with superglue to either end of the chip, both ends were capped with syringe needles, and a 1 ml syringe was attached to one end. Twenty-five microliters of plasma were drawn onto the microarray chip. An alternating current (AC) electric field was applied to the chip for 10 minutes at 14 volts peak-to-peak and 15 kHz to immobilize extracellular vesicles and other nanoparticles onto the microelectrode edges. With the AC field still on, the chip was then washed with 200 uL of 0.5X PBS for an additional 10 minutes. Following the wash step, the AC field was turned off.

### On-chip immunofluorescent labeling and detection of proteins

Because both Tau (a microtubule-associated protein) and GFAP (a filament protein) are likely to be localized within the EV lumen, membranes were permeabilized using 0.1% saponin for 10 minutes. Antibody incubations were for 45–90 minutes at room temperature, or, if recommended by the manufacturer, overnight at 4 °C for optimal binding. For directly conjugated Alexa Fluor 488-anti GFAP antibody (BD Pharmingen), samples were washed with PBS, then visualized and photographed for further analysis. For anti-Tau (Life Technologies), following the wash, Alexa Fluor 594-conjugated secondary antibody (Novex, Life Technologies) was incubated for an additional 90 minutes at room temperature. Following an additional wash, samples were viewed on the microarray chips using an Olympus BX51W epifluorescence microscope with a 4X objective and imaged with Olympus software. All image acquisition parameters were the same for the same fluorophore.

### Quantification of fluorescently-labeled biomarkers

To quantify relative levels of fluorescent antibody-labeled tau or GFAP for each sample, photographic images of each microarray chip were imported to ImageJ (“FIJI”; National Institutes of Health). A circle was drawn around each of eight electrodes, and pixels measured. Background subtracted was the minimum number of pixels measured for each electrode, and averages and standard deviations were calculated. The direct 3D representations of the images were created using the “3D interactive viewer” plug-in for ImageJ.

### Statistical analysis

Logistic regression models were used to predict a diagnosis of glioblastoma in the validation cohort. Separate models were constructed for GFAP and Tau. In each model, the outcome was a diagnosis of glioblastoma and the sole predictor was a binary variable specifying either GFAP rIF > 1.7 or Tau rIF > 2.6. Models were used to predict the probability of a diagnosis of glioblastoma and patients exceeding a specified probability threshold were defined as test positive. The probability threshold was chosen as that which minimizes the Euclidean distance from point (0, 1), or the upper-left corner, on the receiver operating characteristic (ROC) curve. Model predictions were compared to observed diagnoses and performance metrics were calculated, including sensitivity, specificity, positive predictive value (PPV), negative predictive value (NPV), and the area under the ROC curve (AUC). All tests were two-tailed and p < 0.05 was regarded as the threshold for significance. All analyses were carried out using open-source statistical analysis software (R version 3.5.0).

### Disclosure

The contents of this manuscript have not been copyrighted or published previously and are not under consideration for publication elsewhere. All authors agree that the contents of this manuscript will not be copyrighted, submitted, or published elsewhere while acceptance by the journal is under consideration.

## Data Availability

The datasets generated during and analyzed during the current study are available from the corresponding author on reasonable request.
